# Condylar Reshape in Orthognathic Surgery: Morphovolumetric and Densitometric Analysis Based on 3D Imaging and Digital Workflow

**DOI:** 10.1007/s12663-022-01689-3

**Published:** 2022-02-09

**Authors:** Vincenzo Abbate, Giovanni Audino, Giovanni Dell’Aversana Orabona, Marco Friscia, Paola Bonavolontà, Carmelo Lo Faro, Umberto Committeri, Carlos Navarro Cuéllar, Giorgio Iaconetta, Luigi Califano

**Affiliations:** 1 Maxillofacial Surgery Unit, Department of Neurosciences, Reproductive and Odontostomatological Sciences, University Federico II, Via Pansini 5, 80100 Naples, Italy; 2 Maxillofacial Surgery Department, Hospital General Universitario Gregorio Marañón, Madrid, Spain; 3 Neurosurgery Unit,, Department of Medicine, Surgery and Odontoiatrics, University of Salerno, Via Giovanni Paolo II 132, 84084 Fisciano, , Salerno Italy

**Keywords:** Condylar reshape, Digital workflow, Orthognathic surgery, Bone density, Morphovolumetric analysis

## Abstract

**Background:**

Condylar remodelling (CR) is a complex of phenomena that generates in response of the temporo-mandibular joint to forces and stress to maintain a morphological, functional and occlusal homeostasis. The most worrying aspect of the condylar reshape is the condylar resorption which implies fast loss of vertical dimension (>6% of pre-surgical value), mandibular retraction and open bite with preserved articular function.

**Materials and Methods:**

Six parameters were analysed to study the condyles of twelve patients that underwent orthognathic surgery. The digital workflow was then described to make it reproducible enabling a more in-depth study of the reshaping processes that involving the condyle after a great stress like the surgery.

**Results:**

The results of our study showed many statistically significant variations of the studied parameters. In all patients, it was noticed a decreased bone density (*p* = 0,002 per side).

**Objectives:**

The aim of our study, with the aid of the contemporary 3D imaging and digital modelling and workflow technologies, is to investigate and analyse quantitatively and qualitatively the adaptative processes occurring in CR following bimaxillary repositioning. To the best of our knowledge, this is the only paper that investigates the CR considering six different variables at once.

## Introduction

Condylar remodelling (CR) is a group of phenomena that generates in response to forces and stress acting on the temporo-mandibular joint in order to maintain a morphological, functional and occlusal homeostasis. Many factors may influence negatively the reshape capacity as age, sex, systemic pathologies and hormones. [1–3] It has been widely described in literature that female and young patients have bigger CR capacity and that these phenomena also depend on PTH and steroid blood levels. Besides, the main promoting factor is joint compression. This condition may follow orthognathic surgery, orthodontic treatment, bruxism and clenching, occlusal tilt and mandibular trauma. All of these conditions may increase the biomechanical stress on the glenoid fossa. Whenever the mechanical stress overwhelms the adaptive capacities, the condyle suffers pathological changes such as TMJ degeneration and condylar resorption. [4, 5] While joint degeneration results in pain, limited mouth opening and joint noise; condylar resorption implies fast loss of vertical dimension (greater than 6% of pre-surgical value), mandibular retraction and open bite with preserved articular function. In literature, it is possible to assess the incidence of TMJ disorders following orthognathic surgery between 6.7% and 25%. [6–8] Condylar resorption incidence rate varies between 1 and 31%. [9–11] Behind this resorption much have been written, it has been hypothesised that this may be due to the activity of periostine, cytokines, oxygen free radicals and osteoclasts secondary to increased biomechanical stress. [12, 13] Furthermore, Jung et al., Arnett et al., and Mercurio et al. hypothesised that CR following orthognathic surgery may be caused by temporary devascularisation and denervation due to a wide periosteal elevation. [4, 14, 15] Moreover, the prolonged use of elastic orthodontic forces, as well as muscular tension originated from the pterygoid-masseter sling reposition, may influences the joint stress. [16] The aim of our study is to investigate and analyse quantitatively the adaptative processes occurring in CR following bimaxillary repositioning. To the best of our knowledge, this is the only paper that investigates the CR considering six different adaptative variables at once.

## Materials and Methods

Between January 2019 and September 2019, a retrospective chart review was conducted analysing the clinical database of the Maxillofacial Surgery Department of the University Hospital “Federico II” of Naples. All data from patients affected by Angle’s class II and class III malocclusion that underwent orthognathic surgery were collected. In Table 1, there are listed the adopted inclusion and exclusion criteria. Twelve patients met all the criteria to be included in this study. All the selected patients underwent bimaxillary reposition after orthodontic presurgical decompensation treatment. They all underwent weekly follow-up for the first month after surgery and then at 3, 6 and 12 months. All the patients were studied via a preoperatory cone beam computed tomography (CBCT) (T0) and a post 12-months CBCT (T1). Extracting the digital imaging and communications in medicine (DICOM) files, the data of six main variables were collected: height, intercondylar angle, condylar axis inclination, articular surface morphology (anterior, posterior, medial, lateral and superior), condylar volume and bone density (calculated in Hounsfield Unit HU). The data obtained were analytically compared to quantify the CR phenomena.

**Table 1:** Inclusion and exclusion criteria

Inclusion criteria	Exclusion criteria
Angle II and Angle III class malocclusion	Severe facial asymmetries
Bimaxillary orthognathic surgery	Preoperative TMJ pathologies
Preoperative and postoperative orthodontic treatment	Past facial traumas
Older than 18 y.o	Younger than 18 y.o
Preoperative (T0) and 1 year postoperative (T1) CBCT executed by the same machine	Incomplete data

### Data Collection

All the CBCT scans at T0 and T1 were executed by the same operator on the same machine with a field of view (FOV) of 15 × 15 cm. Variables data taken in analysis, from both sides, have been calculated and collected following a reproductible workflow as described after.

### Condylar Height

**Fig. 1:**
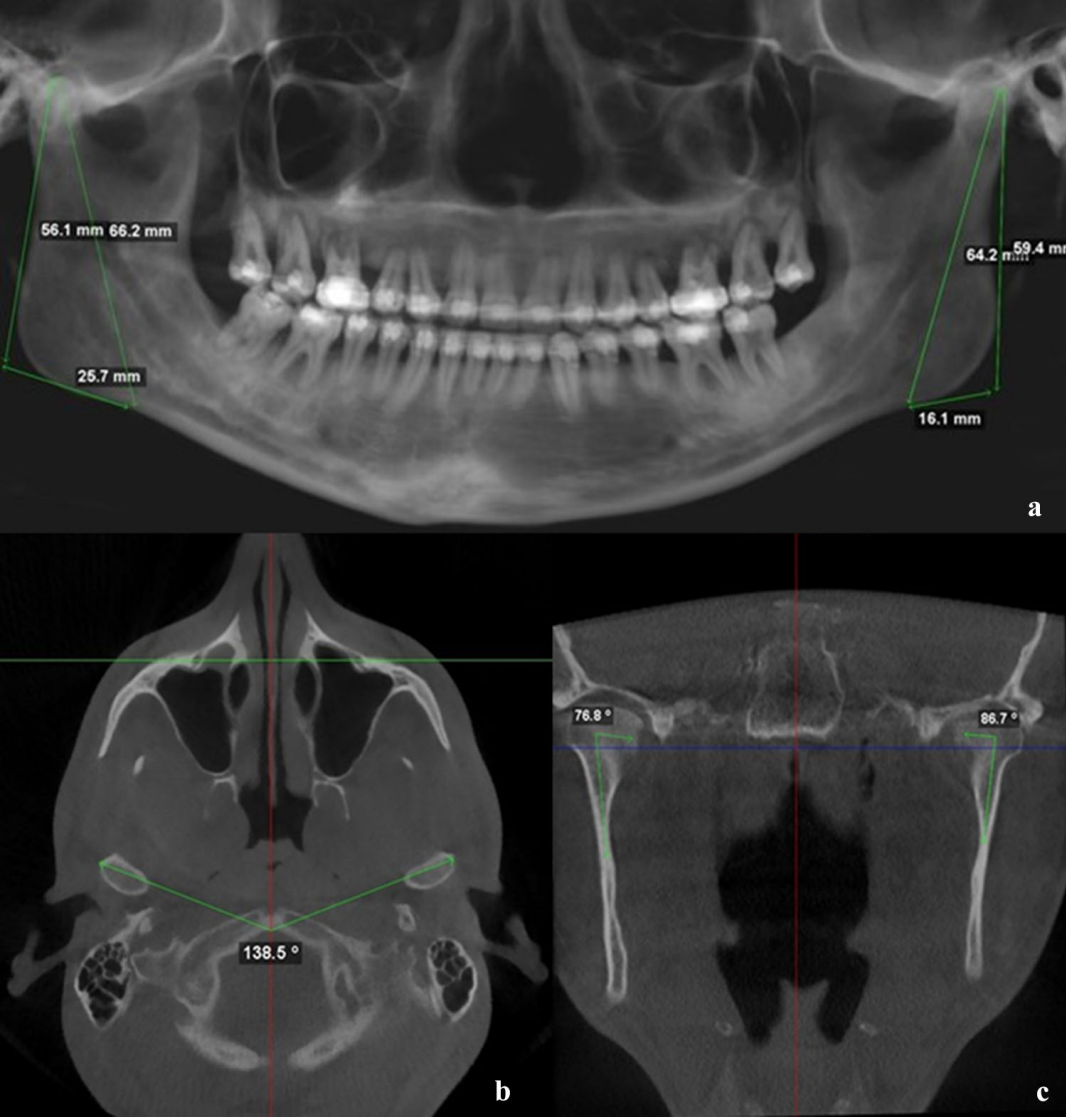
Ramus height measurement (**a**); Intercondylar angle measurement (**b**); Condylar axis measurement (**c**)

DICOM files were imported in Dolphin® software, Dolphin Imaging and Management Solutions Version 11.9 (Chatsworth, CA, USA). Height measure was accomplished via “Build X-Ray” function, followed by “Digitalize/Measure” and “2D line” drawing a tangent line to the ramus from the highest point of the condyle head to the lowest point of mandibular angle, as described by Hoppenreijs (Fig. 1a). [17]

### Intercondylar Angle and Condylar Axis

Using the functions described earlier, it was possible to acquire the intercondylar angle drawing 3 points: A and B (centre of the maximum transverse condylar diameter on left and right side) and vertex (anterior border of magnum foramen) (Fig. 1b). On coronal slices, two lines were drawn, line A (parallel to the maximum transverse diameter) and line B (ramus major axis), the junction of these two lines in the centre of the condyle head is the vertex. This allowed to calculate the condylar axis inclination (Fig. 1c).

### Articular Surface Morphology

**Fig. 2:**
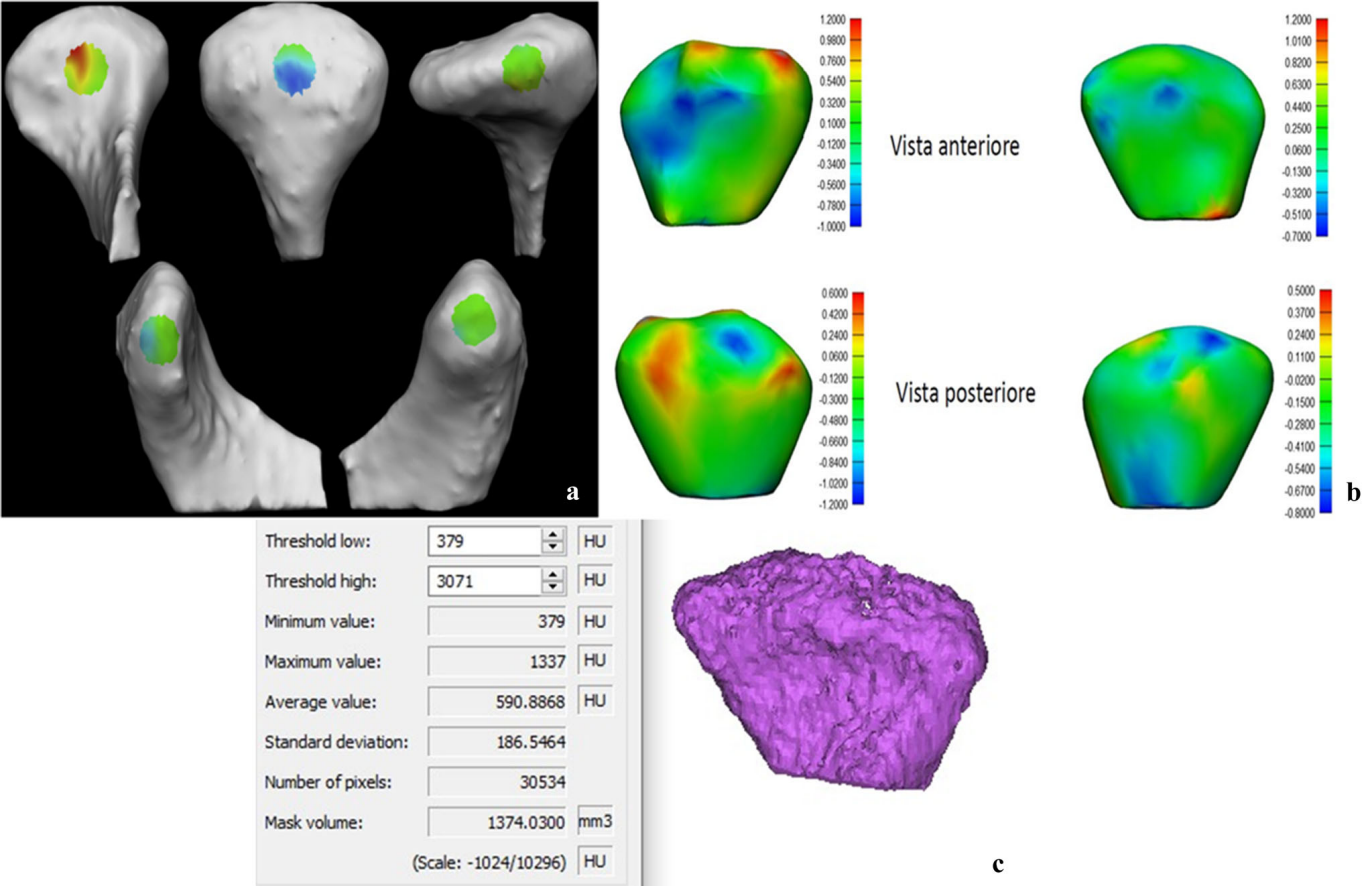
Condylar surfaces (**a**) and morphological variations after volume overlay: resorption areas in blue, neo-osteogenesis area in red (**b**); Condylar density and volume measurement (**c**)

The pre- and postoperative.STL files obtained were then imported on 3-MATIC (Materialise, Leuven, Belgium), retextured and remeshed considering 5 surfaces (anterior, posterior, lateral, medial and superior). On these areas, bone resorption (coloured in blue) and bone apposition (coloured in red) were evaluated to define the morphological modification of the condyle (Fig. 2a, b).

### Condylar Volume and Mean Bone Density

The volume was measured from the highest edge of the condyle to an arbitrarily chosen point 15 mm lower on the condyle axis. The DICOM file was then imported on MIMICS 21.0 software (Materialise HQ Technologielaan, Leuven, Belgium) to manually segment the region of interest and overlay it on the automatic segmentation. This process elaborated the condylar volume as well as its mean density (Fig. 2c).

### Surgical Procedure

Surgical planning was accomplished by the same surgical team via Dolphin® software, Dolphin Imaging and Management Solutions Version 11.9 (Chatsworth, CA, USA), importing CBCT DICOM files, gypsometric modells and facial scans.STL files. The surgical simulation was planned based on cephalometric study. Every patient underwent bimaxillary reposition via Le Fort type I osteotomy and Epker’s BSSO accomplished by the same operator.

### Statistical Analysis

All the collected data were analysed via IBM SPSS Statistics software ver. 28.0 on Windows OS (SPSS Inc., Chicago, IL). Wilcoxon Signed-Rank test for each variable was calculated, sorted per side when possible considering statistically significant *p* value < 0.05.

## Results

In this study,12 patients met all the inclusion criteria, 8 men and 4 women, for a total 24 condyles examined. Mean age was 25.4 ± 5.3 years, ranging from 19 to 37. Ten patients were affected by Angle’s class III malocclusion and two by Angle’s class II malocclusion. Each patient underwent bimaxillary orthognathic surgery via Le Fort I osteotomy and mandibular BSSO. The following surgical procedure were performed: in Angle’s class II malocclusion patients, a mandibular advancement (mean 8.5 ± 6.36 mm) and maxilla impaction (mean 4.5 ± 3.53 mm) while, in class III malocclusion patients, the surgery consisted in maxilla advancement (mean 5.6 ± 1.58 mm) and mandibular setback (mean 7.6 ± 2.21 mm). (Table 2) No surgical complication were assessed after surgery in all the cases. Mandibular ramus height variation was mean −1,02% (mean −0.7 ± 0.8 mm ranging between −3.1 mm and + 2.4 mm, right side *p* = 0,18 and left side *p* = 0,09). Condylar resorption (loss of condylar height) was observed in 18 condyles out of 24 (75%) with a mean reduction of −2,04% of the initial height. (Table 3) The intercondylar angle variations were between −22.6° and + 5.2° (mean −10.8° ± 8.22°), *p* = 0,005. In 2 patients out of 12 (16.6%), it was noticed an increase of the intercondylar angle with condylar extra-rotation. The 83.4% of the patients showed intrarotated condyles following a decreased intercondylar angle. (Table 3) Mean condylar axis variations were + 6.9° ± 8.9° on the right side (*p* = 0,02) and + 1.05° ± 6.71° on the left side (*p* = 0,3). A decreased right condylar axis inclination occurred in only one patient while the left axis inclination decreased in almost the 41.6% of the patients. (Table 3) Seven condyles out of 24 (29%) underwent a mean volume loss of −2.4% ± 2.98%. (Table 3) Most of condyles (71%) gained 3.6% of volume after surgery. In all patients, occurred a decreased bone density (*p* = 0,002 per side). Mean bone density decrease was of 33.74 ± 4.45%: 32.8% in class III dysmorphic patients and 36.6% in class II dysmorphic patients. (Table 3) No statistically significant differences in bone density variations between male (33.8%) and female (33.6%) populations were observed. Colour-maps showed condylar morphological changes affecting the different areas on the articular surfaces. From the analysis, it was possible to notice a greater neo-osteogenesis on anterior (mean 0.09 ± 0.65 mm, right side *p* = 0,26 and left side *p* = 0,13) and medial (mean 0.11 ± 0.51 mm, right side *p* = 0,31 and left side *p* = 0,63) surfaces than on lateral surface. The posterior surface was the most involved in bone re-absorption (mean 0.03 ± 0.55 mm, right side *p* = 1 and left side *p* = 0,5). However, condylar shape data were not statistically significant at t-test (*p* ≥ 0.05). Table 4 summarises all the *p* value sorted per variables and side. In postoperative, antibiotics and steroid therapy were administered for 5 days, cooling face mask for the first 48 h after surgery, and the patients were discharged 3 days after surgery. Not a patient claimed articular pain or discomfort and no adverse events were recorded in immediate and latter postsurgical follow-up.

**Table 2:** Demographic data and surgical procedures accomplished

Patient	Age	Follow-up (months)	Genre	Dysmorphism	Mandibular Advancement (mm)	Maxilla Advancement (mm)	Mandibular Setback (mm)	Maxilla Impactment (mm)
1	27	12	M	Class III	\	5	7	\
2	37	14	M	Class III	\	6	9	\
3	20	15	F	Class III	\	4	6	\
4	22	13	M	Class III	\	5	6	\
5	25	15	F	Class III	\	6	8	\
6	32	12	F	Class III	\	7	9	\
7	21	12	M	Class III	\	5	7	\
8	25	13	F	Class II	8	\	\	4
9	23	13	M	Class III	\	6	8	\
10	32	13	M	Class III	\	7	9	\
11	19	12	M	Class III	\	5	7	\
12	22	12	M	Class II	9	\	\	5
Mean	25,41	13			8,50	5,60	7,60	4,50

**Table 3:** Resulting condylar variations following the surgical treatment

Patient	Condyle Height Variation (mm)		Intercondylar Angle Variation	Condylar Axis Variation		Condylar Volume Variation	
	Right Side	Left Side		Right Side	Left Side	mm^3^	Percentage
1	+ 1.2	−3.1 (−3,89%)	+ 0.5 º	+ 3.3º	+ 5.9º	−42	−2.3%
2	−1.8 (−2,58%)	−0.9 (−1,31%)	−1.7 º	+ 5.3º	−7.0º	8	+ 0.5%
3	−1.2 (−1,81%)	−0.4 (−0,61%)	−13.7 º	−1.6º	+ 1.4º	54	+ 3.1%
4	−2.8 (−3,75%)	−1.4 (−1,95%)	−13.5 º	+ 0.9º	+ 3.6º	148	+ 9.9%
5	−1.2 (−1,81%)	+ 2.4	−7.5 º	−9.3º	−0.3º	22	+ 1.2%
6	−0.8 (−1,12%)	−2.4 (−3,38%)	−13.7 º	+ 1.2º	+ 7.6º	20	+ 1.6%
7	−2.3 (−3,03%)	−1.9 (−2,48%)	+ 5.2 º	+ 6.5º	+ 4.8º	34	+ 1.7%
8	−0.8 (−1,21%)	+ 0.6	−22.6 º	+ 11.3º	+ 7.7º	51	+ 2.7%
9	+ 2.1	−2.7 (−3,18%)	−17.4 º	+ 20.5º	+ 8.2º	69	+ 4.10%
10	+ 1.7	−0.6 (−0,72%)	−11.5 º	+ 17.2º	−5.0º	104	+ 5.6%
11	−1.4 (−1,58%)	−1.1 −1,27%)	−11.8 º	+ 9.7º	−1.0º	26	2%
12	−0.8 (−1,01%)	+ 1.1	−22.0 º	+ 18.5º	−13.3º	29	+ 1.7%
Mean ± sd	−0.6 ± 1.4	−0.8 ± 1.5	−10.80 º ± 8.22	+ 6.95º	+ 1.05º	+ 43.5	+ 2.65%

Patient	Condyle Density Variation (HU)						Condylar Surface Variation (mm)									
	Right Side			Left Side			Anterior		Posterior		Medial		Lateral		Superior	
	^Pre^	^Post^	Δ (%)	^Pre^	^Post^	Δ (%)	R	L	R	L	R	L	R	L	R	L
1	524,6	406,5	−22.5%	498,80	308,60	−36.1%	−1.00	−0.51	+ 0.42	−0.67	00.00	−0.30	−0.56	00.00	+ 0.98	−0.80
2	567,5	376,3	−33.7%	455,80	297,20	−34.8%	+ 0.25	+ 0.60	−1.00	00.00	+ 0.10	+ 0.20	−0.95	−0.80	−0.95	+ 0.32
3	571,1	425,1	−25.6%	351,10	226,50	−35.5%	−0.60	−0.40	+ 0.24	−0.38	00.00	+ 0.50	−1.04	+ 0.80	+ 0.24	−0.44
4	487,7	348,3	−28.6%	492,20	303,60	−38.3%	−0.80	−0.43	−0.85	−0.24	00.00	−0.50	+ 0.35	+ 0.67	+ 1.0	00.00
5	759,5	511,0	−32.7%	580,80	370,60	−36.2%	+ 1.00	+ 0.47	+ 1.18	−0.50	+ 1.50	00.00	00.00	+ 0.46	−0.20	+ 0.58
6	663,5	429,6	−35.2%	539,60	395,50	−26.7%	−0.40	−0.50	−0.50	+ 0.38	+ 0.40	00.00	+ 0.3	−0.39	+ 0.30	+ 0.49
7	380,8	236,8	−37.8%	372,70	223,80	−38.9%	−0.70	+ 1.46	+ 0.40	+ 0.54	+ 0.80	00.00	00.00	00.00	−0.46	−1.00
8	751,8	466,4	−38%	651,90	397,20	−39.1%	+ 0.48	+ 0.99	+ 0.60	−0.50	−0.55	−0.20	00.00	00.00	+ 0.36	−0.69
9	556,9	383,6	−31.1%	602,20	406,90	−32.4%	00.00	+ 0.77	00.00	−0.29	−0.70	+ 0.36	+ 0.70	−0.40	+ 0.75	+ 0.58
10	453,4	295,7	−34.8%	403,30	269,30	−33.2%	+ 0.56	+ 0.65	−0.69	+ 0.80	−0.56	+ 0.65	+ 0.36	−0.51	+ 0.52	00.00
11	511,1	318,2	−37.7%	492,60	336,30	−31.7%	−0.33	−0.34	+ 0.60	−0.44	+ 0.69	−0.60	+ 1.00	+ 1.40	−0.40	−0.34
12	736,3	506,9	−31,1%	698,30	431,00	−38,2%	+ 0.63	+ 0.36	−0.02	+ 0.20	+ 0.57	+ 0.50	−0.33	−0.70	+ 0.68	−0.85
Mean ± sd			−31,4%			−35,1%	+ 0.09 ± 0.65		−0.03 ± 0.55		+ 0.11 ± 0.51		+ 0.01 ± 0.61		+ 0.02 ± 0.61

**Table 4:** *p* value sorted by variables and side

Variable	*p* value	Right side	Left side
Height		*p* = 0.18	*p* = 0.09
Intercondylar angle	***p* = 0,005**		
Condylar axis		***p* = 0,02**	*p* = 0,30
Condylar volume		*p* = 0,46	*p* = 0,11
Bone density		***p* = 0,002**	***p* = 0,002**
*Surface*			
Anterior		*p* = 0,26	*p* = 0,13
Posterior		*p* = 1	*p* = 0,5
Medial		*p* = 0,31	*p* = 0,63
Lateral		*p* = 0,95	*p* = 0,81
Superior		*p* = 0,31	*p* = 0,24
			***p* < 0,05**

Statistically significant values are indicated in bold

## Discussion

CR that follows orthognathic surgery stress includes condylar position variations as well as quantitative and qualitative bone variations. These variations may anticipate the condylar resorption.

### Ramus Height

Hoppenreijs et al. defined as diagnostic parameter for condylar resorption a mandibular ramus height reduction greater than 6% of pre-surgical value. [17] These implies mandibular retreat, loss of posterior vertical dimension and consequent open bite. [18, 19] In literature, condylar resorption is settled between 1 and 31% of cases. [9, 10] Bouwman, Kerstens and Tuinzing noticed a condylar resorption incidence after bimaxillary orthognathic surgery equal to 3% on a pool of 1000 patients. [20] Scheelinck et al. studied this phenomenon in relation to mandibular advancement observing that patients that underwent a mandibular advancement between 5 and 10 mm were subjected to a risk of condylar reabsorption 5 times higher, rising to 20 times higher with advancement greater than 10 mm. [21] Kobayashi et al. collected similar data in six patients that underwent a mandibular advancement greater than 12.1 mm. [18] From these researches, it is common idea to set the cut-off for increased risk of condyle resorption for mandibular advancements greater than 10 mm. Condylar resorption does not seem to be related to mandibular setback. [22] In our pool of patients, not a case of condylar resorption able to cause clinical alterations such as open bites or malocclusion recurrences was observed. Mean ramus height variation was of −0.7 ± 0.8 mm (–1,2%).

### Articular Surface Morphology

In the latter years, many authors adopted computer assisted technologies to improve and to study the morpho-volumetric alterations inducted by orthognathic surgery. [23] Hwang et al. studied the pattern of condylar resorption and neo-osteogenesis in 30 patients affected by class III malocclusion. They found that the anterior and medial surfaces were the most involved in neo-osteogenesis, mean + 0.14 mm and + 0.1 mm, respectively. The opposite for the lateral and posterior surfaces that faced mainly bone resorption, mean −0.19 mm and −0.17 mm, respectively. [24] Similar results were found by Claus et al., that they observed a greater resorption of the posterior surface of the condyle and a greater neo-osteogenesis in the anterior surfaces in a pool of 28 patients that underwent mandibular advancement. [25] Moreover, it has been investigated condylar resorption related to mandible advancement in Class II malocclusion patients showing a strong correlation. [26] In a recent literature review, it has been esteemed that different fixation methods does not influence the condylar reshaping. [27] The obtained results are in line with literature. In our pool of patients, it was observed a neo-osteogenic phenomenon affecting mainly medial and anterior surfaces, 0.11 ± 0.51 mm and 0.09 ± 0.65 mm, respectively. In contrary, posterior surfaces showed mean bone resorption of −0.03 ± 0.55 mm. However, these results did not reach the statistic significancy.

### Intercondylar Angle and Condylar Axis

A statistically significant mean variation after surgery of the intercondylar angle of −10.8° ± 8.22° (*p* = 0,005) as well as a statistically significant mean condylar axis variations + 6.9° ± 8.9° on the right side (*p* = 0,02) and + 1.05° ± 6.71° on the left side (*p* = 0,3) were observed.

### Condylar Volume

Xi et al. studied a pool of 56 patients affected by Angle’s class II malocclusion and compared condylar volumes variations on CBCT scans with the aid of 3D software, finding a volume reduction of the 55% of the condyles, in 65% of cases the patient was a female. [19] These results led the authors to set condylar volume reduction equal to the 17% of the starting value as cut-off to predict an increased risk of resorption and surgical resurgence. It was noticed a volumetric reduction in 7 condyles out of 24 (29%), mean alteration −2.4% ± 2.98%, only 1 condyle of those seven belonged to a female patient. On the other side, 17 condyles out of 24 (71%) met an increase in volume (*p* > 0,05).

The contrast between our results and the results present in literature may be due to the latency between pre-surgery and post-surgery data evaluation. Probably, the volume increase is an early phenomenon that is followed by a reduction.

### Bone Density

Very few studies in literature made out an evaluation of bone density after orthognathic surgery. Nicolielo et al. studied a population of 20 patients that underwent bimaxillary surgery and observed condylar-remodelling phenomenon in 95% of the patients with a mean reduction of bone density equal to 26.4%. They did not notice any correlation to sex and age and the condylar variations. [28] In our study, it is noticeable how in every patient was observed a statistically significant reduction in bone density, meanly 33.74 ± 4.45% (*p* = 0,002 per side). This phenomenon appeared to be related to the class of malocclusion, in fact, class III patients faced a mean density loss of 32.8% while class II patients suffered a mean density reduction of 36.6%. The relation between sex genre and bone density reduction showed not to be statistically significant (33.8% in male patients and 33.6% in female patients). In summa, no condylar resorption case with malocclusion resurgence was noticed, as well as no variation in ramus height greater than 6% of the preoperative data and little morpho-volumetric alteration, in line with literature. The main adaptive mechanism adopted in response to orthognathic surgery was a reduction of bone density, greater in class II patients. The reasons of these variations may be related to an inadequate blood supply to the condyle following the sagittal split. [29, 30] The density reduction may be the earlier adaptive phenomenon in response to orthognathic surgery but its study was possible only recently with the development of modern imaging techs and 3D software. The digital workflow described let us to measure and to compare precisely the condylar adaptation, guaranteeing the reproducibility.

## Conclusion

The variety of adaptive mechanisms of the condyle in response to a great surgical stress, as the orthognathic surgery, is wide, and it includes variation position on transversal, coronal and sagittal plane as well as quantitative and qualitative variation of the bone. In this study, it is presented a digital protocol that, comparing CBCT scans and 3D digital modells, let us to evaluate morpho-volumetric variations as well as densitometric. The results of this research showed that these alterations, involving the mandibular condyles following orthognathic surgery in patients without TMJ diseases or dysfunctions, represent a natural adaptive response and only occasionally they determine a pathologic condition. This adaptive mechanism consists mainly in a reduction of bone density, especially in class II patients, and in a morphologic reshape of condyle surfaces, with bone apposition on medial and anterior surfaces contrasting the bone resorption affecting lateral and posterior surfaces. Our research has for sure some limitations. First of all, it is essential to know how to use at its best the software to reproduce correctly the analysis method. Moreover, these software have a steep learning curve that may require many engineering skills to the surgeons. Nonetheless, the small pool of patients may represent a limit of our study but it is our opinion the reproducibility of our digital workflow makes it replicable on a bigger pool and for a longer follow up giving greater data in order to understand better the condylar adaptive phenomenon.
